# Impact of artificial intelligence (AI) on predicting marginal fit and aesthetic outcomes for custom implant abutments

**DOI:** 10.6026/973206300213962

**Published:** 2025-10-31

**Authors:** Sarathchandra Govind Raj, Venkata Raghavan, Rahul Sharma, Arunagiri Karunanithi, Suma Janya, Minu Raju

**Affiliations:** 1 Department of Prosthodontics, Rajas Dental College and Hospital, Kavalkinaru Junction, Tirunelveli-627105, Tamil Nadu, India; 2 Department of Periodontics, Chettinad Dental College & Research Institute, Kelambakkam, Kanchipuram -603103, Tamil Nadu, India; 3 Department of Oral Medicine and Radiology, Maharana Pratap Dental College and Research Centre, Gwalior, Madhya Pradesh-474006, India; 4 Department of Periodontics, Mahatma Gandhi Postgraduate Institute of Dental Science, Indira Nagar, Gorimedu, Puducherry, India; 5 Department of Prosthodontics, Faculty of Dental Sciences, RUAS (Ramaiah University of AppliedSciences), MSRIT Educampus New BEL Road, Bengaluru-560064, India; 6 Department of Prosthodontics, Mar Baselios Dental college, Kothamangalam, Ernakulam, Kerala-686691, India

**Keywords:** Artificial intelligence, dental implants, marginal fit, custom abutments, aesthetic outcomes, deep learning

## Abstract

The integration of artificial intelligence into implant prosthodontics enhances the precision of pre-fabrication predictions for clinical success. In this study, a custom convolutional neural network (CNN) model achieved a prediction accuracy of 93.5% for marginal fit within a 25 µ tolerance and 87.6% concordance with clinical evaluations of custom implant abutment aesthetics. The AI-estimated mean spatial gap (78.6±18.2 µ) closely approximated the actual measurement (82.3 ± 21.5 µ), with strong correlations observed between predicted and actual outcomes for both fit (r = 0.89) and aesthetic appeal (r = 0.82-0.85). Thus, we show the potential of AI as a preventive quality assessment tool in implant prosthodontics, capable of minimizing adjustments and remakes while improving overall clinical success rates.

## Background:

The success of implant-supported prosthodontics is significantly dependent on how well the marginal fit is between the implant components and the prosthetic structures [1]. Ideally, the better the adaptation, the greater the stability of the prosthesis, which will minimize bacterial microleakage and provide optimal protection for the peri-implant tissues [2]. When adhering to this ideal, marginal gap of exceed 120 µm are correlated with chronic peri-implantitis and then marginal bone loss, where marginal adaptation is a leading cause of biological and mechanical prosthesis failure [3]. Historically, the assessment of marginal fit has typically involved conventional techniques such as direct inspection, radiographic imaging or replica techniques, which are typically subjective, destructive, or have limited diagnostic scope [4]. With the recent adoption of digital workflows and CAD/CAM technology, there has been an increase in the reproducibility and accuracy compared to conventional methods of casting [5]. Yet there is still variability from scanner resolution, milling accuracy and material shrinkage, that introduces variability in the marginal fit, suggesting that while the technology has improved, it is still limited [6]. Aesthetic challenges add yet an additional layer of complexity, particularly to the anterior region where prosthetic contours and gingival harmony play a pivotal role in the overall satisfaction of patients [7]. As mechanically predictable prosthetic components are thoroughly adapted to the situ, any minor design aberration that challenges the molde (abutment) can alter the esthetic result. Artificial Intelligence (AI) has provided a transformational tool in dentistry; most notably, deep learning models, including convolutional neural networks (CNNs), have shown promise in several areas, including imaging diagnosis, treatment planning and prosthetic design [8]. In the area of implant dentistry, AI has been touted as being "highly effective" in accurately identified margin detection, positioning of the implant and identifying challenges or errors. Therefore, it is of interest to develop and evaluate an AI model to predict marginal gaps and esthetic outcomes of custom implant abutments prior to fabrication, thereby shifting quality control from reactive detection to proactive prevention.

## Materials and Methods:

## Study design and sampling:

This study followed 40 patients who were distributed as 24 females and 16 males with an average age of 51.7±13.2 years old. The patient selection process included five essential inclusion criteria which were: (1) age above 18 years, (2) good health status (ASA I or II), (3) sufficient bone support without major reconstruction needs and (4) presence of natural teeth adjacent to and opposing the implant site and (5) follow-up commitment. The patient selection criteria had four main limitations: uncontrolled systemic illnesses, head and neck region radiation history, daily smoking in excess of 10 cigarettes and uncontrolled periodontal conditions and insufficient space between teeth for restorations.

## Implant placement and digital workflow:

The patients received standard two-piece NobelActive titanium implants from Nobel Biocare according to manufacturer guidelines for placement. The patients underwent digital impression capturing with Primescan by Dentsply Sirona after a healing period that required 3 months for mandibular implants and 4 months for maxillary implants. The digital files went through Dental System (3Shape, Copenhagen, Denmark) for the design of patient-specific abutments. The design process included the creation of three different abutment configurations with changing profiles and positioning angles of the marginal complex. A system of virtual designs was anonymized for secure storage in a central database to perform AI analysis and production.

## AI model development and training:

A convolutional neural network CNN utilized ResNet-50 structure but adapted it to process three-dimensional data. The model received its initial training using 10,000 public dental images before undergoing fine-tuning with 500 previously made custom abutments that contained precise marginal fit measurements along with aesthetic scoring. The AI model accepted input features that comprised (1) three-dimensional mesh data of the designed abutment and (2) peri-implant tissue profile from intraoral scans as well as (3) emergence profile parameters and (4) margin location relative to the gingival zenith and (5) abutment angulation. The model received two key output predictions which included expected marginal gap measurements in micrometers and aesthetic outcome scores (PES and WES). The training utilized 80% of data for building the model while validating with 20% and conducted 5-fold cross-validation optimization of hyperparameters. The final predictive model succeeded in reaching an accuracy of 92.3% for identifying marginal gaps within the 30 µm range and 85.7% accuracy for aesthetic score assessment.

## Fabrication and measurement:

Producers randomly selected one virtual design out of three for transformation into actual pieces in each implant location. A 5-axis milling machine (DWX-52DCi, Roland DGA, Irvine, CA, USA) manufactured titanium abutments (Ti-6Al-4V) from blocks. The zirconia crowns received fabrication to fit onto the designed custom abutments.

## Two methods evaluated the implant marginal fit:

[1] The space between implant platform and abutment required measurement through silicone replica method which utilized light-body polyvinyl siloxane. The authors made length measurements at 12 pre-defined points by using a digital microscope at 100x magnification to analyze buccolingual and mesiodistal sections of the samples.

[2] A subset of micro-CT analysis was conducted on 20 abutments using the SkyScan 1172 (Bruker, Kontich, Belgium) micro-CT system with 8µm resolution to generate three-dimensional data of their marginal gaps.

Aesthetic outcomes received judgments from three prosthodontists who utilized the PES/WES system. The evaluators scored the teeth separately after they were installed before capturing images for research analysis.

## Statistical analysis:

Research defined a necessary sample of 60 implants to achieve 80% power for estimating a 15 µm difference in marginal gap sizes at a 0.05 significance level. The researchers analyzed the information through SPSS version 28.0 (IBM Corp., Armonk, NY, USA). An assessment for normality distribution was conducted using the Shapiro-Wilk test method. AI prediction values received statistical analysis through paired t-tests and Wilcoxon signed-rank tests versus actual measurement values. The connection between actual and predicted data was examined by computing Pearson's correlation coefficient. The investigator used ICC to determine the reliability of aesthetic scoring among exam participants. Statistical significance was set at p<0.05.

## Results:

The AI model showed high performance in predicting marginal fit and aesthetic results of custom implant abutments, producing an overall accuracy of greater than 93.5%. The model predicted marginal gap values within 25 µm of actual measurements in 93.5% of cases, with the mean absolute error of 12.7 ± 8.3 µm. When compared to silicone replica measures, there was no statistically significant difference regarding the AI predicted marginal gap (78.6 ± 18.2 µm) versus actual (82.3 ± 21.5 µm, p = 0.089). Micro-CT analysis of 20 abutments supported the model's validity, as the marginal gap measured (79.8 ± 19.7 µm) and the AI predicted (77.4 ± 17.9 µm) showed no significant deviation (p = 0.412). When stratified by implant position, anterior implants had smaller mean values than premolars, which was correctly predicted by the AI. Correlation analysis indicated a strong positive relationship between the AI predicted and actual values (r = 0.89, p < 0.001) and when classifying values as clinically acceptable or unacceptable, Austin's model yielded a sensitivity of 94.2% and specificity of 91.7%. For the aesthetic ratings, we found that AI-predicted scores for both Pink Aesthetic Scores (PES) and White Aesthetic Scores (WES) matched our clinical scores closely with no statistically significant differences between the parameters. The overall AI predicted scores for PES (8.32 ± 1.83) and WES (8.89 ± 1.62) were slightly lower than clinical scores (8.56 ± 1.97 and 9.11 ± 1.58, respectively), with agreement maintained at a high level of 87.6% between PES and WES. The highest level of agreement made by the AI compared to the clinical scores was for surface texture (93.3%), while the lowest agreement was for distal papilla (85.0%). When correlating AI scores with clinician scores, it demonstrated a strong correlation with r = 0.82 for PES, r = 0.85 for WES (both p<0.001) to support predictive accuracy. Inter-rater reliability among our prosthodontists showed an excellent ICC of 0.87 for PES and 0.90 for WES. Interestingly, we noted that aesthetic prediction accuracy was higher for anterior implants (91.3%) than premolars (83.9%) and that PES results were significantly better when testing marginal fit of the abutment were at or ≤80 µm and these findings were corroborated by the AI model. The final validated model could predict accurate results for custom implant abutment marginal fit and aesthetic results at greater than 93.5% accuracy on average. Specifically, the model successfully predicted marginal gap values 93.5% of the time when the predicted results were within 25 µm of the actual measurements. The mean absolute value error between the actual marginal gaps and predicted marginal gaps was 12.7 µm with a standard deviation of 8.3 µm. Table 1 (see PDF) shows the actual values of marginal gaps in addition to AI-generated predictions. The marginal gap predictions from the AI model generated a mean value of 78.6±18.2 µm but showed slight differences to the silicone replica technique measurements at 82.3±21.5 µm. The recorded difference in marginal gap measurements lacked statistically important distinction (p=0.089). About 20 abutments taken for Micro-CT analysis demonstrated an actual mean marginal gap of 79.8±19.7 µm which showed no meaningful difference compared to the AI predictions (77.4±17.9 µm) for these particular abutment samples (p=0.412).

The distribution of marginal gaps across different implant positions showed that anterior implants (incisors and canines) had slightly smaller gaps (mean: 76.4±19.2 µm) compared to premolar implants (mean: 84.1±22.8 µm). This pattern was correctly predicted by the AI model, which estimated mean gaps of 73.8±17.5 µm and 81.9±20.3 µm for anterior and premolar implants, respectively. Analysis of the correlation between predicted and actual measurements showed a strong positive correlation (r=0.89, p<0.001), indicating good predictive validity of the AI model. Furthermore, when categorizing the marginal gaps as clinically acceptable (≤120 µm) or unacceptable (>120 µm), the AI model achieved a sensitivity of 94.2% and specificity of 91.7% in correctly classifying the abutments. Table 2 (see PDF) presents the comparison between AI-predicted aesthetic scores and clinical evaluations by prosthodontists. Aesthetic outcomes were evaluated using the Pink Aesthetic Score (PES) and White Aesthetic Score (WES), each ranging from 0 to 10, with higher scores indicating better aesthetics.

The AI model generated predictions for total PES which were slightly lower at 8.32+/-1.83 compared to clinical evaluations at 8.56+/-1.97 yet their statistical difference remained insignificant (p=0.183). The WES measurement from the AI model assessment (8.89±1.62) demonstrated a small reduction when compared to clinical evaluation results (9.11±1.58) although this difference was not statistically significant (p=0.145). The comparison between AI predictions and clinical assessments yielded an agreement of 87.6% where surface texture received the highest match at 93.3% and the distal papilla produced the lowest match at 85.0%. The relationship analysis showed that AI-predicted scores matched well with clinical examination results as the correlation coefficient measured 0.82 (p<0.001) for PES and 0.85 (p<0.001) for WES. A high level of reliability was detected between the three prosthodontists who conducted data collection (ICC=0.87, 95% CI 0.81-0.92 for PES; ICC=0.90, 95% CI 0.85-0.94 for WES). The computer model displayed superior accuracy in determining the aesthetic results for anterior implants with 91.3% match rate compared to 83.9% accuracy for premolar implants. Cases with marginal gaps less than 80 µm achieved significantly superior PES scores (mean: 9.12±1.21) than those with gaps exceeding 80 µm (mean: 7.68±1.82) and the AI model accurately detected this relationship. Table 1 (see PDF) shows the comparison between AI-predicted marginal gap values and those measured using the silicone replica technique across buccal, lingual, mesial and distal surfaces. It also includes overall mean values, differences and p-values, indicating the level of agreement between predicted and actual measurements. Table 2 (see PDF) presents the comparison of AI-generated aesthetic scores with clinical evaluations by prosthodontists, using both the Pink Aesthetic Score (PES) and White Aesthetic Score (WES). It details parameter-wise mean scores, percentage agreement and statistical analysis, demonstrating the consistency between AI predictions and clinical assessments.

## Discussion:

This study indicates that AI can accurately predict both marginal fit and aesthetic outcomes of custom dental implant abutments prior to fabrication and represents a significant development in the field of implant prosthodontics [9]. Marginal fit prediction is crucial because poor marginal adaptation can contribute to microleakage, peri-implantitis and long-term complications related to the prosthesis [10]. The AI model presented in this study accurately predicted marginal gaps, consistently measuring marginal fit within the clinically acceptable range of 60-120 µm and showing concordance with post-fabrication measurements [11]. As a result, design through an AI model may also act as a viable quality assurance step, with the added benefit of ensuring more precision in fit and reliability in clinical settings [12]. In regard to implant aesthetics, the AI predictions of surface characteristics and soft tissue responses were in strong agreement clinically. Accurate prediction of interaction with soft tissue and emergence profile is particularly important for restoration in the anterior region, as patient enjoyment and satisfaction may relate heavily to visual effects [13]. Furthermore, AI can predict site-specific adjustments for design considerations, allowing the practitioner to anticipate issues before fabrication and limiting chairside adjustments and associated costs. This predictability reduces subjectivity associated with evaluating manual results and thus improves the potential for a reproducible and standardized workflow throughout the workflow [14]. Integrating AI into CAD/CAM processes may provide a number of potential practical advantages. First, identifying design flaws early could reduce remakes and adjustments, saving time and materials, for both clinicians and dental laboratories [15]. Second, AI workflows can support communication between prosthodontists and technicians by providing objective, data-driven feedback during the design process. Third, the use of predictive analytics of digital planning may allow for continued personalization of prostheses based upon patient-specific anatomical and aesthetic preferences [16]. Despite these promising results, there are some limitations to accounting for. First, the limited scope of prosthetic designs and small sample size limits the extent to which findings are generalizable limits that were accounted for in the study [17]. Second, the model's predictions were made in the context of one CAD/CAM system and digital workflow and it is uncertain how well the model's predictions would perform within other workflows and across various complex clinical scenarios. Finally, further research is warranted to assess the predictive accuracy of extreme anatomical cases, multi-unit restorations, or potentially difficult soft tissue conditions [18]. In the future, studies should examine expanding the training datasets for AI to include a wider range of implant systems, abutment characteristics and anatomical features specific to the patient. Longitudinal studies investigating the relationship between outcomes predicted by AI and the clinical performance at a longer duration including stability of peri-implant tissues and longevity of prosthesis will also be necessary. Integration within other digital methods or tools, for example, intraoral scanners or cone-beam computed tomography, would provide a 3D representation of the patient allowing the potential for improved predictive ability of AI.

## Conclusion:

Antecedent to fabrication the AI model demonstrated 93.5% accuracy in evaluating marginal fit together with 87.6% accuracy in determining aesthetic outcomes for custom implant abutments. AI demonstrates strong prediction effectiveness which shows its ability to enhance precise designs and reliable clinical practice. The integration of this system within clinical environments will establish more efficient procedures that ultimately lead to better patient satisfaction while decreasing remake requirements.
